# Student Intrinsic Motivation for Online Creative Idea Generation: Mediating Effects of Student Online Learning Engagement and Moderating Effects of Teacher Emotional Support

**DOI:** 10.3389/fpsyg.2022.954216

**Published:** 2022-07-13

**Authors:** Li Wang

**Affiliations:** School of Chinese Literature and Media, Hubei University of Arts and Science, Xiangyang, China

**Keywords:** intrinsic motivation, online learning engagement, creativity generation, perceived teacher emotional support, moderated mediation effect

## Abstract

The online creativity generation research is a new field of creativity research. However, very little is known about the specific psychological processes of online idea generation. Against this background, this study explored the correlation between student intrinsic motivation and online creativity and possible mechanisms that may lie within this relationship. A sample of 423 Chinese students from three public universities participated in this study by completing measurements of intrinsic motivation, online learning engagement, creativity, and perceived teacher emotional support. The results indicated that student online learning engagement partially mediates the positive association between student intrinsic motivation and their online creativity. Teacher emotional support moderates the positive relationship between student intrinsic motivation and online learning engagement. Our findings suggested that student online creativity benefited from their intrinsic motivation in an online environment. The limitations of this study were also discussed.

## Introduction

The evolution of educational technology and computer-assisted learning has brought many changes to higher education (Seetal et al., 2021). Over the past three decades, online learning has become an important part of the higher education system. One of the main advantages of online learning is that it can provide high-quality educational resources to the public beyond the constraints of time and space. In essence, online learning not only offers unprecedented opportunities to equalize educational resources, but also is preferred by many learners and educators (Roque-Hernández et al., 2021). In particular, the impact of the COVID-19 global health pandemic has forced many higher education institutions to adopt online learning models on a large scale to ensure continuity of instruction (Xin et al., 2020). As a result, online learning is becoming more widely integrated into university learning and teaching systems, which has had a profound impact on the transformation of higher education.

Developing students’ creativity in higher education has been widely recognized, not only because creativity is an important factor in achieving effective and high levels of learning, but also because it is one of the key strategic goals of a nation (Zhang X. et al., 2020). As higher education institutions move from traditional face-to-face models to online learning, research on online creativity has received increasing attention. In previous empirical studies, research on creativity has mainly concentrated on two principal areas: one is associated with individual factors, such as emotional intelligence (Rodrigues et al., 2019), divergent thinking (Gralewski and Karwowski, 2019), openness to experience (Zhang X. et al., 2020), intrinsic motivation (Zhang X. et al., 2020), positive affect (Rego et al., 2014), and autonomous motivation (Liu et al., 2013). Another aspect is related to environmental factors, such as social environments (Carbajal and Baranauskas, 2020), school and family climate (Niu, 2007), cultural background (Jang, 2017), and teacher-student matching (Wang and Qi, 2020). However, until now, it is unclear how individual and situational factors interact in the generation of creative ideas online.

Intrinsic motivation is considered to be an important individual factor in the process of face-to-face creative idea generation (Zhang X. et al., 2020). However, there is limited empirical research on how intrinsic motivation affects online creativity, especially in the context of higher education studies from students’ perspectives. Based on self-determination theory (SDT, Gagn’e and Deci, 2005), which proposes that motivation is consistently associated with more positive relational, performance, and well-being outcomes, this study explores the relationship between student intrinsic motivation and online creativity and possible mechanisms that may lie within this relationship.

## Hypothesis Development

### Student Intrinsic Motivation and Online Creativity

SDT distinguishes between two types of motivation based on different antecedents and consequences of an event (Ryan and Deci, 2000). Intrinsic motivation refers to a type of motivation in which individuals are motivated by the activity itself rather than by the external consequences of their involvement in the task. In contrast, external motivation is generated by external pressures or constraints (Csikszentmihalyi, 2014). According to SDT, internal motivation is considered innate, while external motivation is cultivated through internalization (Fishbach and Woolley, 2022). For example, some students who enter medical school are externally motivated, such as social status and financial gain; while others choose the medical profession for what are considered more intrinsic reasons, such as the desire to save lives and improve society. Having intrinsic motivation is the key to perseverance in the workplace. When people are intrinsically motivated, they treat the work activity as an end in itself, thus making the activity and the goal one and the same. Intrinsic motivation increases interest and enjoyment in the activity. In this study, we defined students’ intrinsic motivation as the enjoyment of learning and the satisfaction of their activity, where the enjoyment is inherent in the learning activity itself (Gottfried et al., 2001).

According to Amabile (1996), improving creativity requires three prerequisites: professional knowledge, creative thinking skills, and intrinsic motivation. Intrinsic motivation plays an important role in creativity. Individuals will not pursue creative activities if they lack intrinsic motivation (da Costa et al., 2015). Although the relationship between intrinsic motivation and creativity is emphasized in Amabile’s theory, empirical research findings on this topic are inconsistent (de Jesus et al., 2013). In some empirical studies, intrinsic motivation appears to be significantly correlated with creative performance. For example, Menges et al. (2017) found that people are driven to perform better if they are interested in what they do. When individuals are intrinsically motivated, they are driven to engage in and work creatively. In contrast, other studies have shown weak or no correlations between intrinsic motivation and creative activity (de Jesus et al., 2013).

Although many empirical studies have pointed out those associations between motivation and creativity across disciplines and levels, there is limited research on the relationship between intrinsic motivation and online creativity in educational contexts. Based on the empirical evidence presented in the previous section, we hypothesize as follows:

*H1*: Student intrinsic motivation has a significant positive effect on their online creativity.

### Mediation Effects of Student Learning Engagement

Bowden et al. (2021) defined student learning engagement as the positive social, cognitive, emotional, and behavioral investments that students make in their interactions with course content, with other students, and with the instructor. Student engagement in learning is an important factor in maintaining students’ connection to the curriculum in educational settings. It is associated with “effective learning” and “deep learning” (Ramsden, 2003). In an online learning environment, student engagement is particularly important. Because online learning cuts down on face-to-face interactions, students often feel isolated and disconnected (Dixson, 2015). Therefore, the effectiveness of online learning needs students’ engagement. Intrinsic motivation helps predict student engagement (Malik et al., 2020). According to the SDT model, engagement is central to motivation (Ryan and Deci, 2000). Conceptual and empirical research suggests that individuals’ intrinsic motivation positively influences affective engagement, which can predict the development of behavioral engagement (Dixson, 2015). In essence, intrinsic motivation leads to deep learning for students.

Student learning engagement helps to stimulate students to have creative ideas. Engaged students are dedicated, enthusiastic, and want to accomplish tasks in innovative ways. In other words, the positive emotions experienced by engaged individuals raise their level of awareness and promote their sense of novelty. Empirical evidence shows that individuals’ engagement can support students in providing new insights in their learning. For example, Zhang X. et al. (2020) emphasized that when students actively engaged in learning, they asked questions and discovered new ideas. Reid and Solomonides (2007) also explored the relationship between engagement and creativity. They found that learning engagement predicted an individual’s level of creativity. Thus, we propose that:

*H2*: Student online learning engagement has a mediation effect on the relationship between student intrinsic motivation and their online creativity.

### Moderating Effects of Teacher Emotional Support

Jiang et al. (2018) divided teacher support into three components: information support, tool support, and emotional support. Information support emphasizes teachers’ provision of information related to learning content; tool support refers to teachers’ provision of learning tools to help students; and emotional support emphasizes teacher’ care, encouragement, and trust in students when they encounter difficulties and stress in the learning process. Specifically, teacher emotional support includes teachers’ concern and respect for students, encouraging students to express their opinions and feelings, and allowing students to make independent decisions (Ruzek et al., 2016). It is an important element of high-quality teaching and learning.

Online learning has many advantages, such as it spans time and space, provides students with a diverse learning environment, and enables resource sharing. However, online learning may also bring some problems such as indifference between teachers and students and a decreased willingness to collaborate (Li et al., 2021). Unlike traditional face-to-face learning environments, the facial expressions, body language, intonation, and other emotional cues of the teacher and students cannot be fully conveyed in an online learning environment, which can lead to negative emotions such as boredom and burnout (Crow and Murray, 2020; Zhang X. et al., 2020). Therefore, when preparing for online learning, teachers must consider such things as providing students with emotional support (e.g., a sense of belonging to a learning community) to facilitate the learning process (Li et al., 2021).

Motivation research has consistently linked teacher emotional support to student motivation and engagement (Cooper, 2014; Ruzek et al., 2016). Self-determination theory emphasizes that the social environment influences individuals’ cognitive and behavioral outcomes (Deci and Ryan, 2012). In other words, intrinsic motivation and engagement can be facilitated when students’ intrinsic needs for relatedness, competence, and autonomy are supported in the classroom (Ryan and Deci, 2000). Good teacher emotional support is an important external driver for students to translate motivation into behavior and produce positive outcomes. Emotionally supportive teachers provide students with more opportunities for autonomy, more interpersonal connections, and a sense of competence. With the emotional support from teachers, students’ intrinsic motivation has a stronger positive effect on learning engagement. Thus, the following hypothesis is proposed:

*H3*: Teacher emotional support moderates the positive relationship between student intrinsic motivation and online learning engagement, such that this relationship is strong for high (vs. low) emotional support.

## Materials and Methods

### Participants

All research processes were reviewed and approved by the academic ethics committee of the author’s university. The sample consisted of students from three public universities in Hubei province, China. Prior to the survey, the researcher contacted the university administration offices *via* email and asked them to invite their students to participate in this study. The questionnaires were sent through a network platform. The system was automatically refreshed when the participants had completed the questionnaire. In addition, the questionnaire was accompanied by an informed consent form that clearly stated the purpose and process of the study. At the same time, this study emphasized the principles of anonymity and confidentiality of participant information. Participation in the survey was voluntary, and participants could withdraw at any time during the survey period. A total of 471 students responded to the questionnaire. Forty eight questionnaires were excluded because participants took less than 5 s to respond, showing a clear tendency to answer in a hurry. Therefore, there were 423 valid questionnaires with effective recovery of 89.8%. Among the survey, there were 194 males (45.9%) and 229 females (54.1%). In terms of grades, 118 participants (27.9%) were freshmen, 155 participants were (36.6%) sophomores, 105 (24.8%) were juniors and 45 (10.7%) were seniors. In terms of majors, 73 (17.3%) participants majored in economics, 155 (36.6%) participants majored in chemistry, 95 (22.5%) participants majored in engineering, 69 (16.3%) participants majored in management, and 31 (7.3%) participants majored in other disciplines. The demographic information is presented in Table 1.

**Table 1:** Demographic information (*N* = 423).

Variable	Category	Frequency	Percentage (%)
Gender	Male	194	45.9
	Female	229	54.1
Grade	Freshman	118	27.9
	Sophomore	155	36.6
	Junior	105	24.8
	Senior	45	10.7
Major	Economics	73	17.3
	Chemistry	155	36.6
	Engineering	95	22.5
	Management	69	16.3
	Others	31	7.3

### Measurements

The student intrinsic motivation was assessed with an adapted form of the intrinsic motivation inventory by Ryan (1982). It has five items. Sample items include “I’ve enjoyed doing this activity” and “I would describe this activity as very interesting.” The measure is rated on five-point Likert scales ranging from 1 = “strongly disagree” to 5 = “strongly agree.” In the current sample, the Cronbach’s alpha of student intrinsic motivation was 0.852.

The online learning engagement was assessed with a 19-item scale developed by Dixson (2015). The items are distributed in four dimensions, namely, skills, emotion, participation, and performance. Nineteen questions that are scored using a 5-point Likert scale. An example of the items in this scale is “Having fun in online chats, discussions or *via* email with the instructor or other students.” The Cronbach’s alpha of student online learning engagement was 0.899.

The student creativity was assessed with a six-item scale developed by Fischer et al. (2019). Sample items include “I generate ideas on how to optimize knowledge and skills with my project team and “I implement ideas with great persistence.” Participants’ response was recorded using a 4-point Likert type scale from 1 = “never or almost never true of me” to 4 = “Always or almost always true of me.” The Cronbach’s alpha of student online creativity for this study was 0.873.

Teacher emotional support was assessed with an adapted form of Overall et al. (2011). It has eight items. Sample items include “My supervisor behaves warmly toward me when discussing my research and/or any problems I am experiencing” and “My supervisor expresses understanding and empathy when I experience difficulties.” The measure is rated on five-point Likert scales ranging from 1 = “strongly disagree” to 5 = “strongly agree.” The Cronbach’s alpha of teacher emotional support for this study was 0.884.

The results of confirmatory factor analysis show that the overall measurement model including all the four latent variables fits the data well: χ^2^ = 549.245, df = 224, χ^2^/df = 2.452, CFI = 0.920, TLI = 0.910, RMSEA = 0.059 (Hu and Bentler, 1999), with the standardized factor loadings ranging from 0.526 to 0.809.

### Data Analysis

Because the focal constructs in this study are latent variables, we used the structural equation modeling (SEM) to model the relationships among variables and then test our hypotheses. Particularly, we performed two SEM analyses using Mplus 7.4. First, we ran a mediation model to test the total effect of student intrinsic motivation on student creativity and the mediating effect of online learning engagement (H1-H2). Second, we ran a moderated mediation model to test the moderating effect of teacher emotional support on the relationship between student intrinsic motivation and online learning engagement, as well as on the indirect effect of intrinsic motivation on student creativity *via* online learning engagement (H3).

Specifically, we deployed the LMS (latent moderated structural equations) method to estimate the latent interaction effect between student intrinsic motivation (independent variable) and teacher emotional support (moderator; Cheung and Lau, 2017). The nonparametric bootstrapping method was used to examine the significance of the mediating effect of online learning engagement and the moderating effect of teacher emotional support on the mediation, because both effects are the product of two path coefficients (Zhao et al., 2010; Hayes, 2015). Specifically, we used 1,000 bootstrap resamples to calculate the bias-corrected 95% confidence intervals (CIs) for the mediating effect and the moderated mediating effect abovementioned. The focused effects can be justified as significant if their bias-corrected 95% CIs do not contain zero (Preacher and Hayes, 2008).

## Results

### Preliminary Analyses

Table 2 shows the means, standard deviations of and correlations among the variables examined in this study. Student intrinsic motivation is positively correlated with student creativity, providing preliminary evidence for H1. Online learning engagement is positively correlated with student intrinsic motivation and student creativity, offering preliminary evidence for H2. Because these variables were measured by self-report scales, common method bias was checked using Harman single-factor method (Podsakoff et al., 2003). The results showed that seven factors with eigenvalues bigger than one were obtained and the first factor only accounted for 25.308% of the total variance of the scale items, less than the 50% threshold. Therefore, common method bias is not a salient threat in our data.

**Table 2:** Descriptive statistics and correlations.

Variable	M	SD	1	2	3	4
1. Student intrinsic motivation	3.437	1.006	1			
2. Online learning engagement	3.581	0.778	0.386^**^	1		
3. Student creativity	2.820	0.896	0.419^**^	0.404^**^	1^**^	
4. Teacher emotional support	3.246	0.958	0.385^**^	0.253^*^	0.258^**^	1

^*^p < 0.05;

** *p* < 0.01.

### Mediating Effect Analysis

Table 3 displays the results of the mediation testing of online learning engagement. The goodness-of-fit for the mediation model is acceptable: χ^2^ = 379.019, df = 191, χ^2^/df = 1.984, CFI = 0.926, TLI = 0.913, RMSEA = 0.048 (Hu and Bentler, 1999). H1 proposes that student intrinsic motivation can positively predict the level of student creativity. As is shown in Table 4, the total effect of student intrinsic motivation on student creativity is significant and positive (*β* = 0.479, *p* < 0.001). Therefore, H1 is supported.

**Table 3:** Results of the mediation model.

Variable	Mediator	Dependent variable
	Online learning engagement	Student creativity
*Control variables*		
Gender	0.053 (0.075)	−0.126 (0.088)
Grade_1	−0.039 (0.119)	−0.118 (0.143)
Grade_2	0.033 (0.115)	−0.334^*^ (0.138)
Grade_3	−0.086 (0.126)	−0.319^*^ (0.146)
Major_1	0.096 (0.154)	0.316 (0.196)
Major_2	0.105 (0.133)	0.321 (0.177)
Major_3	0.226 (0.141)	0.515^**^ (0.179)
Major_4	−0.046 (0.144)	0.383^*^ (0.190)
*Independent variable*		
Student intrinsic motivation	0.344^***^ (0.046)	0.300^***^ (0.077)
*Mediator*		
Online learning engagement		0.520^***^ (0.116)
*R*-square	0.287	0.403

Unstandardized coefficients are reported with standard errors in parentheses.

* *p* < 0.05;

** *p* < 0.01;

*** *p* < 0.001.

**Table 4:** Direct, indirect and total effect of student intrinsic motivation on student creativity.

Effects	Estimates	Bootstrapped 95% CIs
Direct effect	0.300^***^	[0.163, 0.462]
Indirect effect	0.179^***^	[0.110, 0.287]
Total effect	0.479^***^	[0.358, 0.593]

H2 proposes that online learning engagement mediates the relationship between student intrinsic motivation and student creativity. As shown in Table 3, student intrinsic motivation can positively predict online learning engagement (*β* = 0.344, *p* < 0.001). Online learning engagement can also positively predict student creativity (*β* = 0.520, *p* < 0.001). Furthermore, the bias-corrected bootstrapped 95% CI for the mediating effect does not include zero (95% CI = [0.110, 0.287]), indicating that the examined mediating effect is statistically significant (Zhao et al., 2010). Hence, H2 is supported. Since the direct effect of student intrinsic motivation on student creativity is still significant (*β* = 0.300, *p* < 0.001) in the mediation model, online learning engagement thus partially mediates the positive association between student intrinsic motivation and student creativity.

### Moderated Mediation Effect Analysis

Table 5 presents the results of the moderated mediation model. Since the LMS method does not provide traditional fit indices, we adopted the procedures recommended by Maslowsky et al. (2015) to evaluate the goodness-of-fit of the moderated mediation model. First, we ran a baseline model which excludes the latent interaction term (i.e., IM × ES), and the result showed that this baseline model fits the data well (χ^2^ = 726.731, df = 393, χ^2^/df = 1.849, CFI = 0.917, TLI = 0.908, RMSEA = 0.045). Second, we performed a log-likelihood ratio test to evaluate whether the model fit of the full model (i.e., the moderated mediation model including the latent interaction term) is significantly better than that of the baseline model. As indicated by the results of the log-likelihood ratio test (χ^2^ = 6.118, df = 1, *p* = 0.013), the model fit of the full model is significantly better than that of the baseline model. Therefore, we can conclude that the moderated mediation model is also a well-fitted model (Maslowsky et al., 2015).

**Table 5:** Results of the moderated mediation model.

Variable	Mediator	Dependent variable
	Online learning engagement	Student creativity
*Control variables*		
Gender	0.041 (0.073)	−0.130 (0.088)
Grade_1	−0.052 (0.116)	−0.126 (0.144)
Grade_2	0.046 (0.111)	−0.339^*^ (0.138)
Grade_3	−0.079 (0.122)	−0.322^*^ (0.146)
Major_1	0.146 (0.149)	0.313 (0.196)
Major_2	0.128 (0.125)	0.316 (0.176)
Major_3	0.215 (0.134)	0.504^**^ (0.179)
Major_4	−0.022 (0.134)	0.379^*^ (0.189)
*Independent variable*		
Student intrinsic motivation (IM)	0.310^***^ (0.049)	0.294^***^ (0.078)
*Mediator*		
Online learning engagement		0.546^***^ (0.118)
*Moderator*		
Teacher emotional support (ES)	0.120^*^ (0.053)	
*Latent interaction*		
IM × ES	0.168^**^ (0.058)	
*R*-square	0.367	0.410

Unstandardized coefficients are reported with standard errors in parentheses.

* *p* < 0.05;

** *p* < 0.01;

*** *p* < 0.001.

**Figure 1:**
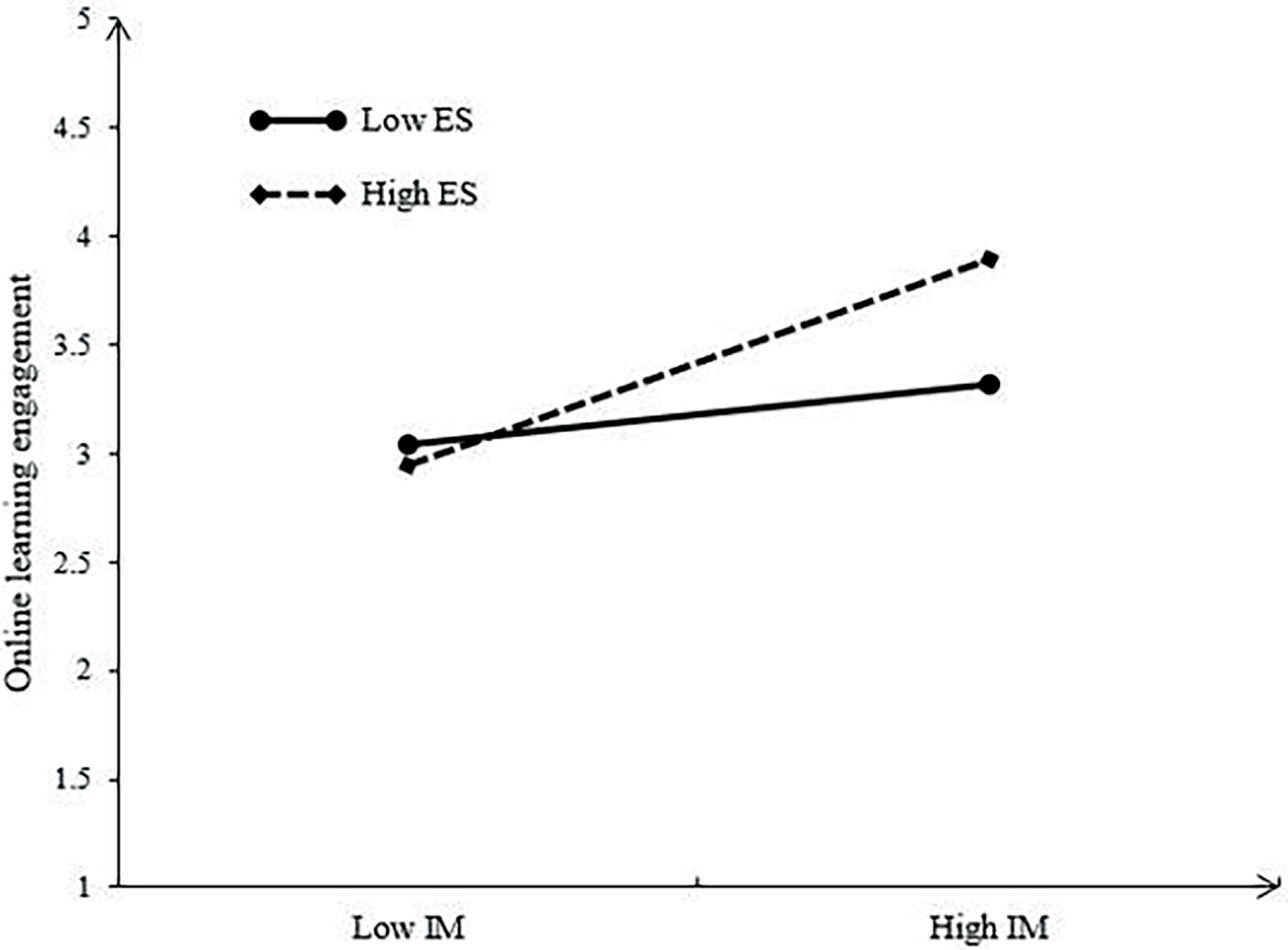
The moderation plot for teacher emotional support. IM, student intrinsic motivation and ES, teacher emotional support.

Table 5 shows that the path coefficient of the latent interaction term (IM × ES) is significant and positive (*β* = 0.168, *p* = 0.004), indicating that the moderating effect of teacher emotional support on the association between student intrinsic motivation and online learning engagement is statistically significant. Simple slope test was further conducted and we found that when students perceive low-level teacher emotional support (M − SD), the path coefficient from student intrinsic motivation to online learning engagement is 0.165, but when students perceive high-level teacher emotional support (M + SD), the path coefficient will reach 0.456. As a result, the positive relationship between student intrinsic motivation and online learning engagement can be stronger for high (vs. low) level of teacher emotional support (see Figure 1), supporting H3.

Furthermore, we tested whether the mediating effect of online learning engagement is dependent on the level of teacher emotional support. The results show that the index of the moderated mediation is significant (index = 0.092, bootstrapped 95% CI = [0.032, 0.189] excluding zero; Hayes, 2015). Thus, teacher emotional support moderates the mediating effect of online learning engagement. Table 6 demonstrates that the mediating effect of online learning engagement is higher when students perceive a high level of emotional support from teachers than when students perceive a low level of emotional support.

**Table 6:** Mediating effect of online learning engagement conditional on teacher emotional support.

Moderator value	Mediating effect	Bootstrapped 95% CIs
M − SD	0.090^*^	[0.021, 0.192]
M	0.169^***^	[0.104, 0.269]
M + SD	0.249^***^	[0.142, 0.385]

M, mean; SD, standardized deviation; and CIs, confidence intervals. *p < 0.05; ***p < 0.001.

## Discussion

### Main Findings

The present study used a moderated mediation to examine whether student online learning engagement would mediate the link between student intrinsic motivation and their online creativity, and whether teacher emotional support would moderate the relationship between student intrinsic motivation and student online learning engagement. Overall, our findings supported our hypotheses.

Consistent with our hypothesis, the present study suggested that student intrinsic motivation, as a major source of dynamism in educational settings, may be an important factor in student online creativity. The current study confirmed and extended previous findings that found intrinsic motivation to be positively associated with creativity (e.g., Tan et al., 2016; Malik et al., 2020). In prior studies, researchers focused on the fact that student intrinsic motivation can promote creativity in face-to-face learning environments (Zhang X. et al., 2020). However, the effect of student intrinsic motivation on their creativity in online environments is unclear. Our findings suggested that student online creativity benefited from intrinsic motivation in an online environment, which supports the findings of Zhang X. et al. (2020).

Another major finding of this study was that student online learning engagement partially mediates the positive association between student intrinsic motivation and their online creativity as the mediation model verification shows. According to the SDT model, individual behaviors are positively correlated with motivation (Ryan and Deci, 2000). Although prior research supports the relationship between student intrinsic motivation and creative performance, the possible mechanisms involved in this relationship are unclear (Eldor and Harpaz, 2016; Yu et al., 2019). To our knowledge, the present study is the first to explore the mediating role of student engagement in online learning between student intrinsic motivation and student creativity. Consistent with our hypothesis, student intrinsic motivation could predict creative performance through the indirect effect of student online learning engagement. In other words, student intrinsic motivation can lead students to deep learning and thus become more engaged in the online learning environment, which in turn stimulates students to generate creative ideas.

Our findings confirmed that teacher emotional support played a moderating role in the influence of student intrinsic motivation on online learning engagement. According to the SDT mode, the external environment can influence an individual’s cognition and behavior by satisfying his or her psychological needs. In educational settings, teacher emotional support is an important factor in teacher-student interaction that facilitates students’ social and emotional functioning and learning. In other words, when students’ intrinsic needs are supported in learning, their intrinsic motivation and engagement are facilitated (Ryan and Deci, 2000). The present study showed that students’ intrinsic motivation had a stronger positive effect on learning engagement when they were emotionally supported by the teacher, which is consistent with the findings of Dixson (2015).

### Limitations and Future Research

We are aware that there may be some limitations in this paper. First, we only assessed a small number of college students from Chinese universities (423 sample data), which may affect the representativeness of the sample compared to the size of online students worldwide. A large sample of students and faculty from different countries and cultural backgrounds could more accurately reveal the impact of key factors on student online creativity.

Another limitation of this study is that many other variables may influence this setting, such as different teachers and teaching methods, physical environment, culture, etc. We did this because, in predicting student creativity online, we were interested in focusing on the role of teacher emotional support in facilitating student intrinsic motivation, student learning engagement, and creativity. The results demonstrated the potential value of this construct in predicting student creativity online, which will provide some theoretical and technical support for online instruction. In future work, we plan to add some influencing factors and try to build a more complete model.

Despite these limitations, we believe the present study can provide an overall understanding of the relationship between instinctual motivation, online creativity, learning engagement, and teacher emotional support. We highlight the importance of teacher emotional support in creating a better creative online environment. We suggest that we need to further investigate the main concepts of this study and expand the sample to reduce the effect of cultural background or age factor on the results. Therefore, we suggest analyzing this possible confounding variable in further research.

## Data Availability Statement

The raw data supporting the conclusions of this article will be made available by the authors, without undue reservation.

## Author Contributions

The author confirms being the sole contributor of this work and has approved it for publication.

## Funding

The completion of this article has been supported by the project of Hubei University of Arts and Science (XK2020021).

## Conflict of Interest

The author declares that the research was conducted in the absence of any commercial or financial relationships that could be construed as a potential conflict of interest.

## Publisher’s Note

All claims expressed in this article are solely those of the authors and do not necessarily represent those of their affiliated organizations, or those of the publisher, the editors and the reviewers. Any product that may be evaluated in this article, or claim that may be made by its manufacturer, is not guaranteed or endorsed by the publisher.
